# Fabrication of UF‐white cheese: Obtaining a different proteolysis rate, texture, and flavor via using combinations of mesophilic starter culture and *Lactobacillus helveticus*

**DOI:** 10.1002/fsn3.3769

**Published:** 2023-10-16

**Authors:** Arash Tondhoush, Mostafa Soltani, Fatemeh Azarikia, Aziz Homayouni‐Rad, Mostafa Karami

**Affiliations:** ^1^ Department of Food Sciences and Technology, Faculty of Pharmacy, Tehran Medical Sciences Islamic Azad University Tehran Iran; ^2^ Nutrition and Food Sciences Research Center, Tehran Medical Sciences Islamic Azad University Tehran Iran; ^3^ Department of Food Technology, Faculty of Agricultural Technology (Aburaihan) University of Tehran Tehran Iran; ^4^ Department of Food Science and Technology, Faculty of Nutrition & Food Sciences Tabriz University of Medical Sciences Tabriz Iran; ^5^ Department of Food Science and Technology, College of Food Industry Bu‐Ali Sina University Hamedan Iran

**Keywords:** *Lactobacillus helveticus*, microstructure, proteolysis, rheological properties, sensory characteristics, UF‐white cheese

## Abstract

The effect of using mesophilic starter culture (*Lactococcus lactis* ssp. *lactis* and *Lactococcus lactis* ssp*. cremoris*) and *Lactobacillus helveticus (L. helveticus)* at different ratios (100:0, 75:25, 50:50, 25:75, and 0:100) on the quality properties of UF‐white cheese during 90 days of ripening was studied. The results revealed that an increase in *L. helveticus* ratio caused a significant decrease in the pH and total protein contents of the cheeses (*p* < .05). No significant changes were observed in the dry matter content of the cheeses (*p* > .05). The use of higher ratios of *L. helveticus* led to a noticeable increase in proteolysis and lipolysis indices in the cheeses (*p* < .05). The cheese produced with higher ratios of *L. helveticus* had less storage (G′) and loss (G″) moduli compared to other cheeses. The more open structure was seen in the cheeses produced using higher ratios of *L. helveticus*. Regarding sensory properties, lower scores of body and texture, and higher scores of odor and flavor were assigned to the cheeses produced using higher ratios of *L. helveticus*. In conclusion, the use of combinations of mesophilic starter culture and *L. helveticus* at specific ratios (75:25 and 25:75) led to improve quality characteristics of UF‐white cheese.

## Graphical abstract

Various ratios of mesophilic starter culture (*Lactococcus lactis* ssp. *lactis* and *Lactococcus lactis* ssp. *cremoris*) and *Lactobacillus helveticus* (100:0, 75:25, 50:50, 25:75, and 0:100) were used for the production of UF‐white cheese. The use of higher ratios of *Lactobacillus helveticus* led to a noticeable increase in proteolysis and lipolysis indices in the cheeses (*p* < .05). The cheese produced with higher ratios of *Lactobacillus helveticus* had less storage (G′) and loss (G″) moduli compared to other cheeses.

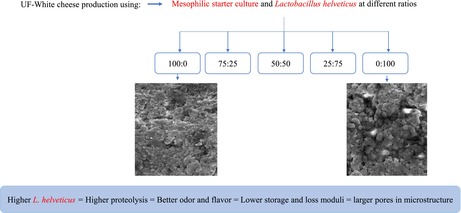

## 1 INTRODUCTION

White cheese is known as semi‐hard cheese with a sliceable compact texture, slightly acidic flavor and white to yellowish color. Various types of milk, i.e., cow, sheep, goat, and buffalo milk, are used for manufacturing this type of ripened cheese, which is commonly preserved in brine before consumption. In the rural production units, ripening time of the cheeses produced from raw milk are 6–8 months; while ripening of white cheese takes 40–90 days in the industrial level. It is well known that white cheese is one of the most consumed cheeses around the world, which is also used to produce various types of processed or local cheeses (Hayaloglu, 2017; Soltani et al., 2022).

For standardizing the production process as well as obtaining a product with approvable quality and industrial‐scale safety, the ultrafiltration technique (UF) is recommended for cheese manufacturing. UF is a method for milk concentration before the formation and handling of the curd, which avoids the removal of whey during the production of cheese. In addition to having higher fat and protein levels, UF cheeses have higher moisture contents in comparison with the cheeses made using conventional techniques. That is due to the high concentration of whey proteins and their great capability of water‐holding capacity. Furthermore, since high concentration of whey proteins inhibits the function of chymosin, slower ripening and flavor development were observed in UF cheeses in comparison with conventional methods (Soltani et al., 2015, 2022).

Deniably, starter cultures have a remarkable effect on the quality properties of the obtained cheeses, i.e., texture, flavor, and microbial safety. Production of acid during the fermentation process, contribution in enzymatic proteolysis and lipolysis stages of the ripening, conversion of fatty acids and amino acids into flavoring compounds, and inhibition of development of undesirable microorganisms because of specific compounds such as organic acids, bacteriocins, and hydrogen peroxide are the most important functions of starter cultures (Coelho et al., 2022; Fox et al., 2017).

The most common starter cultures used for cheese production are the strains of lactic acid bacteria. Among these strains, *Lactobacillus helveticus* (*L. helveticus*) is a thermophilic lactic acid culture with a cell wall‐bound proteinase and intracellular peptidases and lipases. The liberation of intracellular enzymes via autolysis can result in the conversion of amino and fatty acids to aroma compounds. It has been reported that use of *L. helveticus* as primary or adjunct culture during cheese production led to development of flavor, reduction of bitterness, and acceleration of proteolysis in various types of cheeses (Baptista et al., 2018; Cuffia et al., 2018; Sıçramaz et al., 2022; Yang et al., 2021). Along with high acid tolerance and the generation of complex proteolytic enzymes, *L. helveticus* is able to produce lactic acid from glucose and galactose in remarkable amounts. On the other hand, unlike some bacteria, i.e., *Streptococcus thermophiles*, *Lactobacillus acidophilus*, and *Lactobacillus rhamnosus*, which can only metabolize glucose, *L. helveticus* can metabolize both glucose and galactose and produce more acid contents compared to the above‐mentioned bacteria (Chelladurai et al., 2023; Zhang et al., 2022).

Mesophilic starter culture containing *Lactococcus lactis* ssp. *lactis* and *Lactococcus lactis* ssp*. cremoris* are used as common culture in producing UF‐white cheese on industrial scale. To the knowledge of authors, there is no study on the contribution of *L. helveticus* as a starter culture in the production of UF‐white cheese. Our hypothesis was based on the effectiveness of *L. helveticus* in the acceleration of proteolysis and improvement of texture and flavor in UF‐white cheese which is known to have slower proteolysis compared to conventional cheeses because of the inhibition effect of whey proteins on the activity of coagulant enzyme (Chelladurai et al., 2023; Nazari et al., 2020; Soltani et al., 2016, 2022). Thereupon, the objective of this study was to investigate the effect of using various combinations of *L. helveticus* and mesophilic cultures (*Lactococcus lactis ssp. lactis plus Lactococcus lactis* ssp. *cremoris*) on the chemical composition, proteolysis, lipolysis, rheological properties, microstructure and sensory characteristics of UF‐white cheese during 90 days of ripening.

## 2 MATERIALS AND METHODS

### 2.1 Materials

Raw cow milk and all equipment for the retentate production were provided by Pegah dairy plant (Kerman, Iran). Mesophilic homofermentative culture (containing *Lactococcus lactis* ssp. *lactis plus Lactococcus lactis* ssp. *cremoris*, DM‐230) and *L. helveticus* (LH100) were obtained from Danisco Company (Danisco Deutschland GmbH, Niebüll, Germany). *Rhizomucor miehei* protease as milk‐clotting enzyme (Fromase 2200 TL Granualte, ≥2200 international milk clotting units/g) was obtained from DSM Food Specialties (Seclin, Cedex, France).

### 2.2 Cheese‐making procedure

UF‐white cheeses were produced in three separate trials of consecutive weeks in Pegah dairy plant (Kerman, Iran). A UF system with a 5 t/h milk intake capacity was employed to obtain the retentate. The spiral wound‐type membrane (SPIRA‐CEL®‐Modules, MICRODYN‐NADIR, GmbH, Germany) containing three modules and a total surface area of 427 m^2^ were used for the UF process. The cheese production stages continued until obtaining the retentate, according to the method used by Soltani et al. (2015). UF was applied to the milk in 52 ± 1°C and at 140 kPa for 900 s and had the inlet and outlet pressure of 520–540 kPa and 140–160 kPa, respectively. At the end of the UF process, 1 kg retentate was obtained from 5.1 kg milk. After application of heat treatment (78°C, 1 min) and homogenization (5 MPa), 25 kg of obtained retentate was divided into five equal portions; then, various blend of mesophilic lactic acid bacteria and *L. helveticus* were added to the retentate as starter culture (20 g per 1000 kg of retentate), according to the following strategy:

- Cheese A: 100% of mesophilic starter culture +0% of *L. helveticus*.
- Cheese B: 75% of mesophilic starter culture +25% of *L. helveticus*.
- Cheese C: 50% of mesophilic starter culture +50% of *L. helveticus*.
- Cheese D: 25% of mesophilic starter culture +75% of *L. helveticus*.
- Cheese E: 0% of mesophilic starter culture +100% of *L. helveticus*.

The retentate was then held at 35°C until pH reached to 6.3–6.4. Next, *Rhizomucor miehei* protease was added to the retentate (30 g per 1000 kg of retentate). The mixture was filled immediately into containers (100 g) and sent to the coagulation tunnel (35°C, 20 min). Afterward, parchment paper was placed on top of the cheeses and dry salt (3%) was sprayed on the parchment paper. As final stage, the cheese containers were sealed with aluminum foil and held at 26 ± 1°C for 24 h, until the pH reached to 4.7–4.8. The produced cheeses were then transferred to a cold room (9 ± 1°C), and analysis was performed after 1, 30, 60, and 90 days of ripening.

### 2.3 Physicochemical analysis

Cheese samples were analyzed in duplicate for measurement of dry matter using oven‐drying method at 102 ± 1°C (IDF, 1982), fat by the Van Gulik method (Ardo & Polychroniadou, 1999), and the total nitrogen by the micro‐Kjeldahl method (IDF, 1993). For pH measurement, 10 g of grated cheese was mixed with 10 mL of distilled water, and the pH value of the resultant slurry was monitored using a digital pH meter (model SevenCompact S220K, Mettler‐Toledo, Greifensee, Switzerland). The values of titratable acidity (% LA) in the cheeses were determined according to the method described by Manzo et al. (2019) based on titration of diluted cheese samples with NaOH (0.1 N).

### 2.4 Proteolysis

#### 2.4.1 Soluble nitrogen fractions

Water‐soluble nitrogen (WSN) and 12% trichloroacetic acid soluble nitrogen (TCA‐SN) fractions in the cheeses were determined by the method described by Hayaloglu et al. (2005) and expressed as % of total nitrogen. To determine the amounts of total free amino acids (FAA) in the cheeses, the method proposed by Hayaloglu (2007) was utilized.

#### 2.4.2 Urea‐PAGE of caseins

The degree of casein hydrolysis in the cheeses during ripening was monitored using freeze‐dried water‐insoluble fractions of the cheeses and urea‐polyacrylamide gel electrophoresis (Urea‐PAGE) instrument (Protean II XI vertical slab gel unit, Bio‐Rad Laboratories Ltd., Watford, UK) according to the method described by Andrews (1983). Staining of the obtained gels was accomplished directly by the method of Blakesley and Boezi (1977) with Coomassie Brilliant Blue G‐250. Next, the gels were destained by pure water, and gel slabs were digitized using a scanner (HP ScanJet software, ScanJet G4010, Hewlett Packard, Palo Alto, CA).

### 2.5 Lipolysis

For the analysis of lipolysis extent as acid degree value (ADV), the butyrometer containing 10 g of grinded cheese and 20 mL of the reagent (30 g triton x‐100 and 70 g sodium tetra phosphate in 1 L distilled water) was placed in a water bath (90–100°C, 20 min) in order to extract fat. After centrifuging the mixture (2264 g, 1 min) and addition of aqueous methanol for transferring the fat to the neck of the butyrometer, re‐centrifugation in the mentioned condition was implemented. The separated liquid fat was then transferred to an erlenmeyer, weighed, titrated with 0.02 KOH, and ADV was calculated (Case et al., 1985).

### 2.6 Rheological measurements

The rheological experiments of cheeses were accomplished at 25 ± 1°C using a rheometer (Model MCR 301 Anton Paar GmbH, Anton Paar‐Str. 20, Graz, Austria) equipped with parallel plates geometry (PP25), according to method described by Karami et al. (2009). Sample was cut in disc form (25 mm in diameter and 2 mm in height) with a special wire at 9 ± 1°C and sealed with a plastic film in order to prevent of moisture loss. Then, the samples were kept at 25 ± 1°C for 3 h for temperature equilibration. For studying viscoelastic properties, the storage modulus (G') and loss modulus (G") were measured as a function of frequency (0.1–100 Hz). The linear viscoelastic range was obtained by performing a strain sweep test at 0.1 Hz frequency as the percentage of strain values varied from 0.01% to 10.00%, and a strain in the linear region was selected (0.022).

### 2.7 Microstructure

A scanning electron microscopy (XL Series, model XL30, Philips, Netherlands) with 10,000× magnification was used to monitor the microstructure of the cheeses, according to the method described by Madadlou et al. (2006). A layer of gold was utilized to coat the samples. The images were prepared at a voltage of 15 kV, the day after cheese production.

### 2.8 Sensory analysis

Sensory evaluation of the cheeses was implemented after 1, 30, 60, and 90 day of ripening by 9 expert panelists from the Laboratory of Food Analysis at Tehran Medical Sciences, Islamic Azad University (Tehran, Iran) who were familiar with UF‐white cheese. Cheeses were evaluated based on color and appearance (scale 0–5), body and texture (scale 0–5), and odor and flavor (scale 0–10). Moreover, the sum of sensory scores given to cheeses by panelists during ripening was presented as total score (Clark & Costello, 2009). In this regard, half an hour before sensory evaluation, the coded cheeses were removed from the cold room and kept at room temperature. About 100 g cheese was presented to the panelist, and water was also provided for rinsing their mouths between samples (Soltani et al., 2015). A heat map was also prepared for the sensory properties of UF‐white cheeses.

### 2.9 Statistical analysis

The data obtained from three trials were analyzed statistically using the analysis of variance (ANOVA) using SPSS (version 16.0, SPSS Inc., USA). Different groups were statistically compared by Duncan's multiple range tests. Analysis was performed for 1, 30, 60, and 90 days of ripening, and statistical significance was considered at *p* < .05 (Guo et al., 2018).

## 3 RESULTS AND DISCUSSION

### 3.1 Chemical composition of raw milk and cheeses

The mean values for chemical composition of the milk used in the three cheese‐making trials were 12.36 ± 0.11% for DM, 3.30 ± 0.01% for fat, 3.27 ± 0.04% for protein, 6.67 ± 0.01 for pH, and 0.15 ± 0.01 for titratable acidity (as % of lactic acid), which were normal for cow milk as reported by several previous researchers (Khosrowshahi et al., 2006; Mousavi et al., 2023; Soltani et al., 2015). The chemical composition of the cheeses is presented in Table 1. According to obtained results, significant changes were determined in the chemical composition of UF‐white cheeses produced using various starter ratios (*p* < .05). The pH of the cheeses was 4.53–4.66 at the first day of ripening, which was in accordance with the results of previous researches for UF‐white cheese (Mousavi et al., 2023; Soltani et al., 2019; Yousefi et al., 2021). An increase in the *L. helveticus* ratio caused a significant decrease in the pH and a significant increase in the titratable acidity of the cheeses at each sampling time (*p* < .05). Therefore, the cheese E which produced using the highest ratio of *L. helveticus* had the lowest value of pH and the highest value of titratable acidity during ripening (*p* < .05). The higher acidification rate of *L. helveticus* compared to mesophilic starter culture led to accelerating the conversion of lactose to lactic acid, higher dropping the pH, and increase in the titratable acidity in the cheese (Vasiliauskaite et al., 2022). On the other hand, a significant decrease in the pH and a significant increase in the titratable acidity values were observed during ripening in all cheeses, probably due to continuing the conversion of lactose to lactic acid with progressing the fermentation and liberation of free fatty and amino acids via lipolysis and proteolysis phenomena (Mousavi et al., 2023; Rashtchi et al., 2021).

**TABLE 1:** Chemical composition of the UF‐white cheeses produced using different ratios of mesophilic starter culture and *L. helveticus* during ripening (*n* = 3).

	A*	B	C	D	E
pH					
1	4.66 ± 0.01^aA^	4.64 ± 0.01^abA^	4.63 ± 0.01^abA^	4.61 ± 0.02^bA^	4.53 ± 0.02^cA^
30	4.53 ± 0.01^aB^	4.50 ± 0.01^abB^	4.48 ± 0.02^bB^	4.47 ± 0.03^bB^	4.30 ± 0.03^cB^
60	4.45 ± 0.02^aC^	4.42 ± 0.02^bC^	4.39 ± 0.01^bcC^	4.37 ± 0.04^cC^	4.16 ± 0.02^dC^
90	4.39 ± 0.03^aD^	4.37 ± 0.02^abD^	4.34 ± 0.02^bD^	4.27 ± 0.02^cD^	3.98 ± 0.05^dD^
Acidity (% LA)					
1	0.86 ± 0.01^dD^	0.91 ± 0.02^cD^	0.93 ± 0.01^cD^	0.96 ± 0.01^bD^	1.01 ± 0.02^aD^
30	1.03 ± 0.02^dC^	1.09 ± 0.02^cC^	1.12 ± 0.02^bC^	1.20 ± 0.03^aC^	1.21 ± 0.04^aC^
60	1.15 ± 0.02^eB^	1.20 ± 0.04^dB^	1.24 ± 0.03^cB^	1.29 ± 0.02^bB^	1.32 ± 0.02^aB^
90	1.20 ± 0.04^eA^	1.25 ± 0.03^dA^	1.29 ± 0.02^cA^	1.36 ± 0.03^bA^	1.39 ± 0.03^aA^
Dry matter (%)					
1	36.94 ± 0.17^aA^	36.85 ± 0.15^aA^	36.79 ± 0.18^aA^	36.70 ± 0.20^aA^	35.86 ± 0.43^aA^
30	36.43 ± 0.16^aB^	36.40 ± 0.13^aB^	36.37 ± 0.12^aB^	36.29 ± 0.09^aB^	35.76 ± 0.36^aA^
60	35.69 ± 0.20^aC^	35.67 ± 0.17^aC^	35.62 ± 0.21^aC^	35.44 ± 0.25^aC^	34.86 ± 0.09^aB^
90	35.29 ± 0.39^aD^	35.24 ± 0.36^aD^	35.14 ± 0.35^aD^	35.01 ± 0.41^aD^	34.19 ± 0.10^aC^
Fat‐in‐dry matter (%)					
1	51.99 ± 0.19^cA^	52.05 ± 0.14^cA^	52.31 ± 0.16^bA^	52.36 ± 0.24^abA^	52.41 ± 0.30^aA^
30	51.48 ± 0.39^dB^	51.57 ± 0.40^cdB^	51.66 ± 0.45^cB^	51.81 ± 0.61^bB^	52.05 ± 0.24^aB^
60	50.96 ± 0.26^dC^	51.17 ± 0.16^cC^	51.24 ± 0.19^cC^	51.41 ± 0.29^bC^	51.69 ± 0.39^aC^
90	50.68 ± 0.38^eD^	50.87 ± 0.39^dD^	51.03 ± 0.22^cD^	51.20 ± 0.22^bD^	51.40 ± 0.28^aD^
Total protein (%)					
1	13.92 ± 0.06^aA^	13.59 ± 0.19^bA^	13.13 ± 0.14^cA^	12.95 ± 0.16^dA^	12.71 ± 0.12^eA^
30	13.19 ± 0.11^aB^	13.01 ± 0.09^bB^	12.72 ± 0.15^cB^	12.55 ± 0.14^dB^	12.29 ± 0.18^eB^
60	12.56 ± 0.23^aC^	12.36 ± 0.26^bC^	12.19 ± 0.19^cC^	11.95 ± 0.06^dC^	11.46 ± 0.16^eC^
90	11.84 ± 0.26^aD^	11.52 ± 0.08^bD^	11.46 ± 0.06^bcD^	11.30 ± 0.13^cD^	11.06 ± 0.18 ^dD^

*Note*: Means with different superscripts within each UF‐white cheese (upper case) and within each ripening time (lower case) for each of the parameters measured, are different (*p* < .05).

* Cheeses A: 100% mesophilic starter culture; B: 75% mesophilic starter culture + 25% *L. helveticus*; C: 50% mesophilic starter culture + 50% *L. helveticus*; D: 25% mesophilic starter culture + 75% *L. helveticus*; E: 100% *L. helveticus*.

The type and ratio of starter culture had not a significant effect on the dry matter (DM) contents of the UF‐white cheeses (*p* < .05). However, higher acidification rate of *L. helveticus* compared to mesophilic starter cultures inhibited protein–protein interactions, increased moisture content, and slightly decreased DM content of the cheese produced using higher ratios of *L. helveticus* (Sıçramaz et al., 2022). The values of the DM in the present study were similar to the values reported by Karami et al. (2009) and Soltani et al. (2015) and slightly lower than the values determined by Soltani et al. (2019) for UF‐white cheese. On the other hand, fat‐in‐dry matter (FDM) content of the cheese was affected by the various types and ratios of starter culture (*p* < .05). A parallel trend was seen in the changes in DM and FDM contents of the cheeses during ripening. It was reported that DM changes and in consequence FDM changes in cheese are occurred because of diffusion between salt and water molecules in the cheese matrix during ripening (Guinee & Fox, 2017).

The total protein content of UF‐white cheeses was significantly (*p* < .05) influenced by the various mixtures of *L. helveticus* and mesophilic starter cultures used for production. Using higher ratios of *L. helveticus* as starter culture caused a significant decrease (*p* < .05) in the total protein content of the cheeses D and E compared to other cheeses during ripening, probably due to the greater proteolytic activity and higher degradation of protein in the case of using *L. helveticus* compared to mesophilic cultures (Baptista et al., 2018; Cuffia et al., 2018). It has been also reported that proteolysis and diffusion of water‐soluble nitrogen into brine significantly decreased (*p* < .05) the total protein content of all cheeses during ripening (Soltani et al., 2019, 2022). Less or no syneresis during ripening and limited protein loss through the whey drainage because of the absence of curd cutting occur in UF cheeses. However, and despite the limiting role of whey proteins in the proteolytic activity of coagulating enzymes and lactic starters, proteolysis is continued at a slower rate in the UF cheeses compared to conventional ones in the consequence of proteolytic action of the enzymes retained totally in the curd (Mistry & Maubois, 2017).

### 3.2 Proteolysis

#### 3.2.1 Soluble nitrogen fractions

Although remarkable phenomena including glycolysis and lipolysis occurred during cheese ripening, as stated, proteolysis is the most important one which causes desirable development in the texture and flavor of the final product through degradation in protein structure and liberation of short‐chain peptides. In this context, the study of proteolysis indices can help to a better understanding of the changes occurred in cheese quality during ripening (Ardö et al., 2017).

The values of WSN (as % of TN) and TCA‐SN (as % of TN) as a representative of primary and secondary proteolysis in the UF‐white cheeses during ripening are shown in Table 2. The cheeses produced using higher ratios of *L. helveticus* as starter culture (cheeses D and E) had significantly (*p* < .05) higher contents of WSN compared to other cheeses during ripening, due to higher proteolytic activity of *L. helveticus* compared to mesophilic starter culture (Kumar et al., 2015; Sıçramaz et al., 2022). In this regard, cheese A which produced using 100% of mesophilic culture and cheese E which made with 100% of *L. helveticus* had the lowest and highest values of WSN among the cheeses, respectively. It was found that there was a lack of synergy between the activities of the used starter cultures. Although it was expected that proteolysis could be enhanced by using the mixture of two cultures; interestingly, it was found that *L. helveticus* had inhibitory effect on the activity of mesophilic starter culture (Sıçramaz et al., 2022). On the other hand, proteolysis was continued during ripening and reached to the highest level in all cheeses at the end of the ripening which was due to high proteolytic activity of the starter cultures used for cheese production (Soltani et al., 2015, 2019).

**TABLE 2:** Soluble nitrogen fractions of UF‐white cheeses produced using different ratios of mesophilic starter culture and *L. helveticus* during ripening (*n* = 3).

	A*	B	C	D	E
WSN (% of TN)					
1	11.63 ± 0.13^eD^	12.00 ± 0.17^dD^	12.43 ± 0.24^cD^	13.19 ± 0.11^bD^	13.51 ± 0.07^aD^
30	11.92 ± 0.06^eC^	12.32 ± 0.12^dC^	12.70 ± 0.09^cC^	13.41 ± 0.21^bC^	13.88 ± 0.11^aC^
60	12.27 ± 0.12^eB^	12.73 ± 0.14^dB^	13.51 ± 0.18^cB^	13.85 ± 0.34^bB^	14.18 ± 0.48^aB^
90	12.75 ± 0.17^eA^	13.29 ± 0.08^dA^	13.82 ± 0.05^cA^	14.15 ± 0.43^bA^	14.53 ± 0.43^aA^
TCA‐SN (% of TN)					
1	4.76 ± 0.04^eD^	4.95 ± 0.09^dD^	5.20 ± 0.08^cD^	5.41 ± 0.18^bD^	5.60 ± 0.31^aD^
30	5.70 ± 0.07^eC^	5.73 ± 0.05^dC^	6.23 ± 0.07^cC^	6.97 ± 0.09^bC^	7.31 ± 0.21^aC^
60	6.97 ± 0.12^eB^	7.03 ± 0.13^dB^	8.83 ± 0.13^cB^	9.38 ± 0. 14^bB^	10.12 ± 0.22^aB^
90	9.09 ± 0.13^eA^	9.41 ± 0.08^dA^	10.00 ± 0.09^cA^	10.69 ± 0.09^bA^	12.10 ± 0.24^aA^
Free amino acids (mg Leu/g)					
1	5.36 ± 0.04^eD^	6.95 ± 0.09^dD^	8.20 ± 0.08^cD^	9.41 ± 0.18^bD^	10.60 ± 0.31^aD^
30	6.42 ± 0.07^eC^	7.83 ± 0.05^dC^	9.23 ± 0.07^cC^	10.67 ± 0.09^bC^	11.71 ± 0.21^aC^
60	7.87 ± 0.12^eB^	9.03 ± 0.13^dB^	10.53 ± 0.13^cB^	11.48 ± 0.14^bB^	12.52 ± 0.22^aB^
90	9.19 ± 0.13^eA^	10.41 ± 0.08^dA^	12.06 ± 0.09^cA^	12.79 ± 0.09^bA^	13.61 ± 0.24^aA^

*Note*: Means with different superscripts within each UF‐white cheese (upper case) and within each ripening time (lower case) for each of the parameters measured, are different (*p* < .05).

* Cheeses A: 100% mesophilic starter culture; B: 75% mesophilic starter culture + 25% *L. helveticus*; C: 50% mesophilic starter culture + 50% *L. helveticus*; D: 25% mesophilic starter culture + 75% *L. helveticus*; E: 100% *L. helveticus*.

Measurement of nonprotein nitrogen compounds, i.e., TCA‐SN can be suitable for the evaluation of secondary proteolysis products; for instance, urea, small peptides, and amino acids (Solhi et al., 2020). As can be seen in Table 2, increasing *L. helveticus* ratio led to an increase in TCA‐SN contents of UF‐white cheese (*p* < .05). Higher degradation of primary proteolysis products in the presence of *L. helveticus* compared to mesophilic starter culture caused increase in the value of TCA‐SN in the cheeses D and E compared with the cheeses A and B (Hayaloglu et al., 2014; Soltani et al., 2022). Furthermore, a significant increase (*p* < .05) was also determined in TCA‐SN contents in all cheeses due to release of peptides with low or intermediate molecular weight in the result of residual coagulant activity (Barac et al., 2019; Soltani et al., 2019).

The type of starter cultures besides known parameters i.e. coagulating enzyme and ripening conditions are influenced the content of total FAA in cheese (Ganesan & Weimer, 2017; Moreira et al., 2018; Niro et al., 2017; Sıçramaz et al., 2022). Significant changes in the total FAA content of the cheeses were observed via alteration of the ratios of mesophilic starter culture and *L. helveticus* (*p* < .05). The cheese manufactured using 100% *L. helveticus* had the highest value of total FAA. However, during ripening, this value was in the lowest content in the cheese produced with 100% mesophilic culture. Total FAA contents of the UF‐white cheese were increased continuously as increase in the ratio of *L. helveticus* used for cheese production due to noticeable proteolytic activity of *L. helveticus* compared to mesophilic culture as the consequence of higher cell autolysis. Continuous increase in the contents of FAAs as the end products of proteolysis during ripening of cheese expresses the extent and depth of proteolysis. Moreover, conversion of initial curd to a cheese with desirable texture and flavor depends to gradual degradation of proteins and generation of free amino acids. On the other hand, with an increase in the ripening duration, the content of total FAA increased in all cheese samples due to the coagulating enzyme and especially starter proteases activities along with the progress of proteolysis (Khattab et al., 2019; Nazari et al., 2020). The increase in total FAA of the UF‐white cheeses had the same trend as an increment in other proteolysis indices during ripening due to the progress in proteolysis (Table 2).

#### 3.2.2 Urea‐PAGE of caseins

**FIGURE 1:**
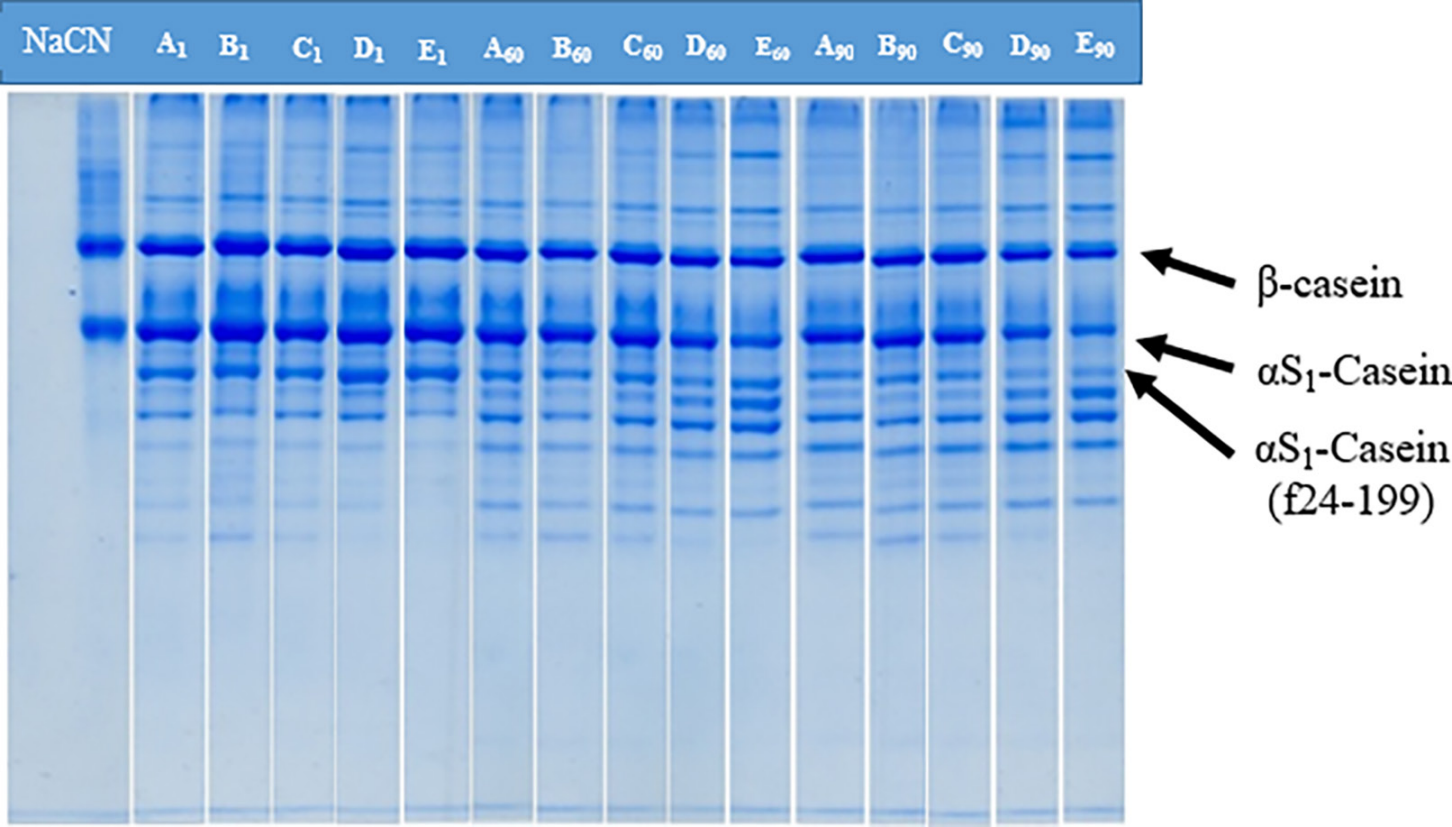
Urea‐PAGE electrophoretograms of the water‐insoluble fragments of the UF‐white cheeses produced using different ratios of mesophilic starter culture and *L. helveticus*, during ripening (Cheeses A: 100% mesophilic starter culture; B: 75% mesophilic starter culture + 25% *L. helveticus*; C: 50% Mesophilic starter culture + 50% *L. helveticus*; D: 25% mesophilic starter culture + 75% *L. helveticus*; E: 100% *L. helveticus*).

Urea‐PAGE is a useful technique for evaluating the status of caseins in the matrix of the cheese at any stages of ripening. Urea‐PAGE electrophoretograms of the water‐insoluble fractions of the cheeses during ripening are presented in Figure 1. Considerable hydrolysis was seen in α_s1_‐CN and β‐CN after 60 days of ripening in the cheeses. In addition, great differentiation was observed in the electrophoretogram patterns of the cheeses during ripening. The degradation level of α_s1_‐CN was higher in the cheeses produced using higher ratios of *L. helveticus* compared to the cheeses produced using higher ratios of mesophilic starter culture. Moreover, the use of higher levels of *L. helveticus* as starter culture ended to higher degradation of α_s1_‐CN (f 24‐199) in the cheeses D and E, compared to other cheeses. This result indicated a higher proteolytic capacity of *L. helveticus* in comparison with mesophilic starter culture, which led to more degradation of α_s1_‐CN to related peptides. More hydrolysis of caseins in the presence of *L. helveticus* have been reported previously for Scamorza, Swiss‐type and Prato cheese (Baptista & Gigante, 2022; Guidone et al., 2015; Skrzypczak et al., 2020). Furthermore, it was reported that lower pH is favorable for the activity of residual coagulant in the cheese matrix in order to hydrolyze α_s1_‐CN. In this regard, more hydrolysis of α_s1_‐CN was observed in cheeses D and E with lower pH values than in other cheeses (Özer & Kesenkaş, 2019).

Changes in the type or ratios of the starter culture influenced the degradation of β‐CN in the cheeses. However, due to more stability against the acidic conditions, the rate of degradation in β‐CN was lower than α_s1_‐CN in the cheeses (Romeih et al., 2002; Soltani et al., 2022). In addition, existence of some inhibitory factors like β‐lactoglobulin can cause a decrease in the degradation rate of β‐CN and formation of hydrolysis products, i.e., γ_1_, γ_2_, and γ_3_ caseins (Gutiérrez‐Méndez et al., 2019; Niro et al., 2021). On the other hand, by progressing the ripening, a decline was observed in the staining of the bands in the electrophoresis patterns of all cheeses which was in accordance with the increase in soluble nitrogen fragments during ripening which indicated the significant effect of ripening on the intensity of proteolysis (Solhi et al., 2020).

### 3.3 Lipolysis

Lipolysis that is defined as the hydrolysis of milk fat through the activity of lipases and esterases has a notable role in the flavor development in cheese during the ripening time. The use of *L. helveticus* alone or in combination with mesophilic culture caused a noticeable increase in lipolysis index of UF‐white cheese (Table 3). As the cheeses produced with higher ratios of *L. helveticus* (cheeses D and E) had considerably higher lipolysis indexes compared to other cheeses during ripening (*p* < .05). It was reported that *L. helveticus* strains have the ability to enhance lipolysis, formation of short, medium‐ and long‐chain fatty acids, and development of flavor in cheese during ripening in order to achieve the desired quality characteristics (Cuffia et al., 2018; Slattery et al., 2010).

**TABLE 3:** Lipolysis index of UF‐white cheeses produced using different ratios of mesophilic starter culture and *L. helveticus* during ripening (*n* = 3).

	A*	B	C	D	E
Lipolysis index (mL KOH 0.1 N/g fat)					
1	4.43 ± 0.09^dD^	4.46 ± 0.07^dD^	6.13 ± 0.09^cD^	7.59 ± 0.11^bD^	8.71 ± 0.07^aD^
30	5.92 ± 0.06^eC^	6.32 ± 0.12^dC^	8.70 ± 0.09^cC^	10.41 ± 0.12^bC^	11.88 ± 0.11 ^aC^
60	7.27 ± 0.12^eB^	8.73 ± 0.14^dB^	11.51 ± 0.18^cB^	13.85 ± 0.14^bB^	15.18 ± 0.16^aB^
90	13.75 ± 0.15^eA^	16.29 ± 0.08^dA^	19.82 ± 0.05^cA^	22.45 ± 0.17^bA^	24.53 ± 0.13^aA^

*Note*: Means with different superscripts within each UF‐white cheese (upper case) and within each ripening time (lower case) for each of the parameters measured, are different (*p* < .05).

* Cheeses A: 100% mesophilic starter culture; B: 75% mesophilic starter culture + 25% *L. helveticus*; C: 50% mesophilic starter culture + 50% *L. helveticus*; D: 25% mesophilic starter culture + 75% *L. helveticus*; E: 100% *L. helveticus*.

According to the results, the lipolysis index was increased at a significant level in all cheeses during ripening (*p* < .05) probably due to bacterial growth and lipolytic activities of different milk components, i.e., principal indigenous lipase and lipoprotein lipase (Estrada et al., 2019).

### 3.4 Rheological analysis

**FIGURE 2:**
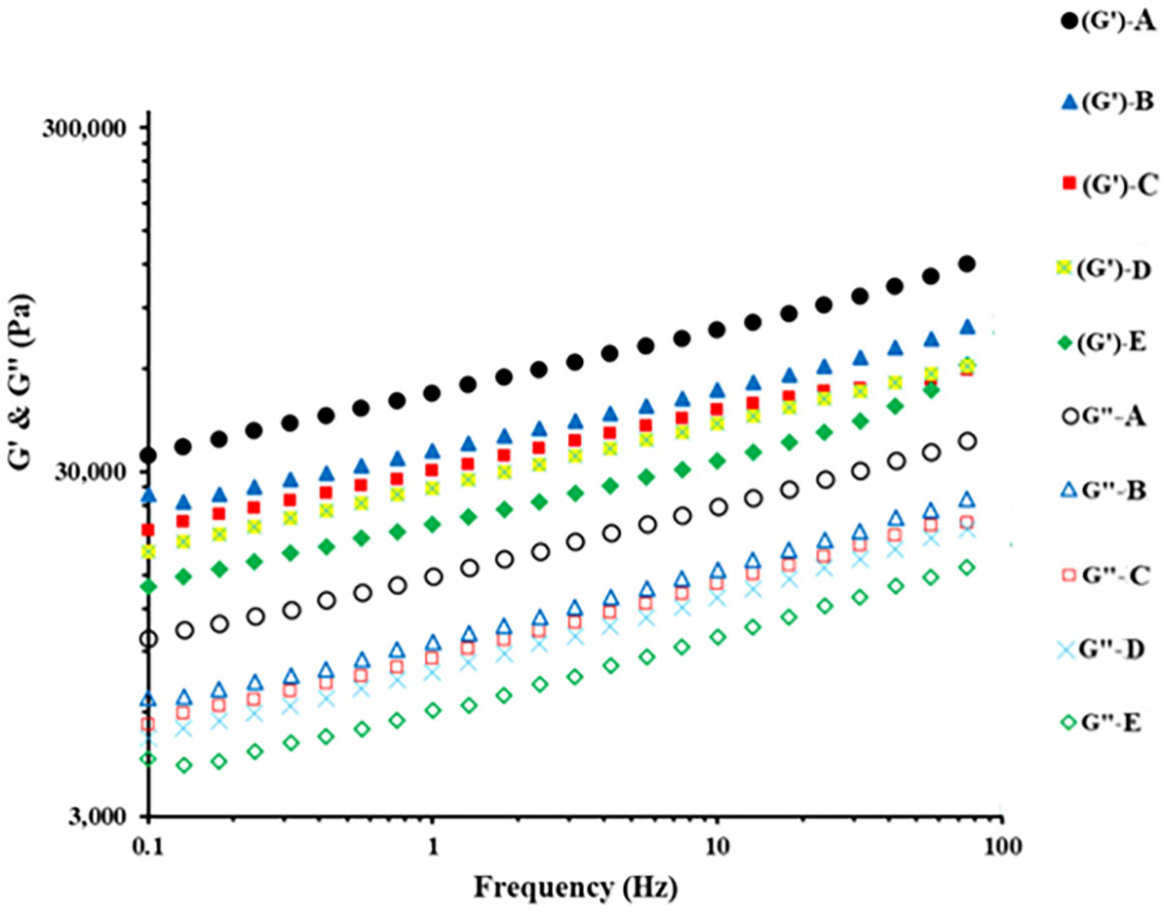
Rheological properties of the UF‐White cheeses produced using different ratios of mesophilic starter culture and *L. helveticus* after 90 days of ripening (Cheeses A: 100% mesophilic starter culture; B: 75% mesophilic starter culture + 25% *L. helveticus*; C: 50% mesophilic starter culture + 50% *L. helveticus*; D: 25% mesophilic starter culture + 75% *L. helveticus*; E: 100% *L. helveticus*).

The most usual method for oscillatory testing is studying the storage (G′) and loss (G′′) moduli as a function of frequency, which exhibits elastic and viscous behaviors of cheese, respectively (Soltani et al., 2016). Alteration of rheological properties of the UF‐White after 90 days of ripening is presented in Figure 2. As can be seen, G′ was greater than G″ in all cheeses, and this situation expresses the domination of elastic behavior to viscous. Similar results have been reported for UF‐white cheese by Karami et al. (2009) and Soltani et al. (2016). On the other hand, the use of different ratios of *L. helveticus* and mesophilic starter cultures affected the rheological properties of UF‐white cheeses. The D and E cheeses that produced using higher ratios of *L. helveticus* had the lowest storage (G′) and loss (G″) moduli at the studied frequency range, probably due to the higher proteolytic activity of *L. helveticus* compared to mesophilic starter culture, higher degradation of proteins, and a decrease in the cross‐links among the nonlinear strands of caseins. In contrast, the A cheeses that produced with 100% of mesophilic culture had the highest G′ and G″ values among the cheeses. It was reported that higher protein content in the cheese may lead to an increase in solid‐like structure and higher firmness (Bekele et al., 2019; Karami et al., 2009).

### 3.5 Microstructure

**FIGURE 3:**
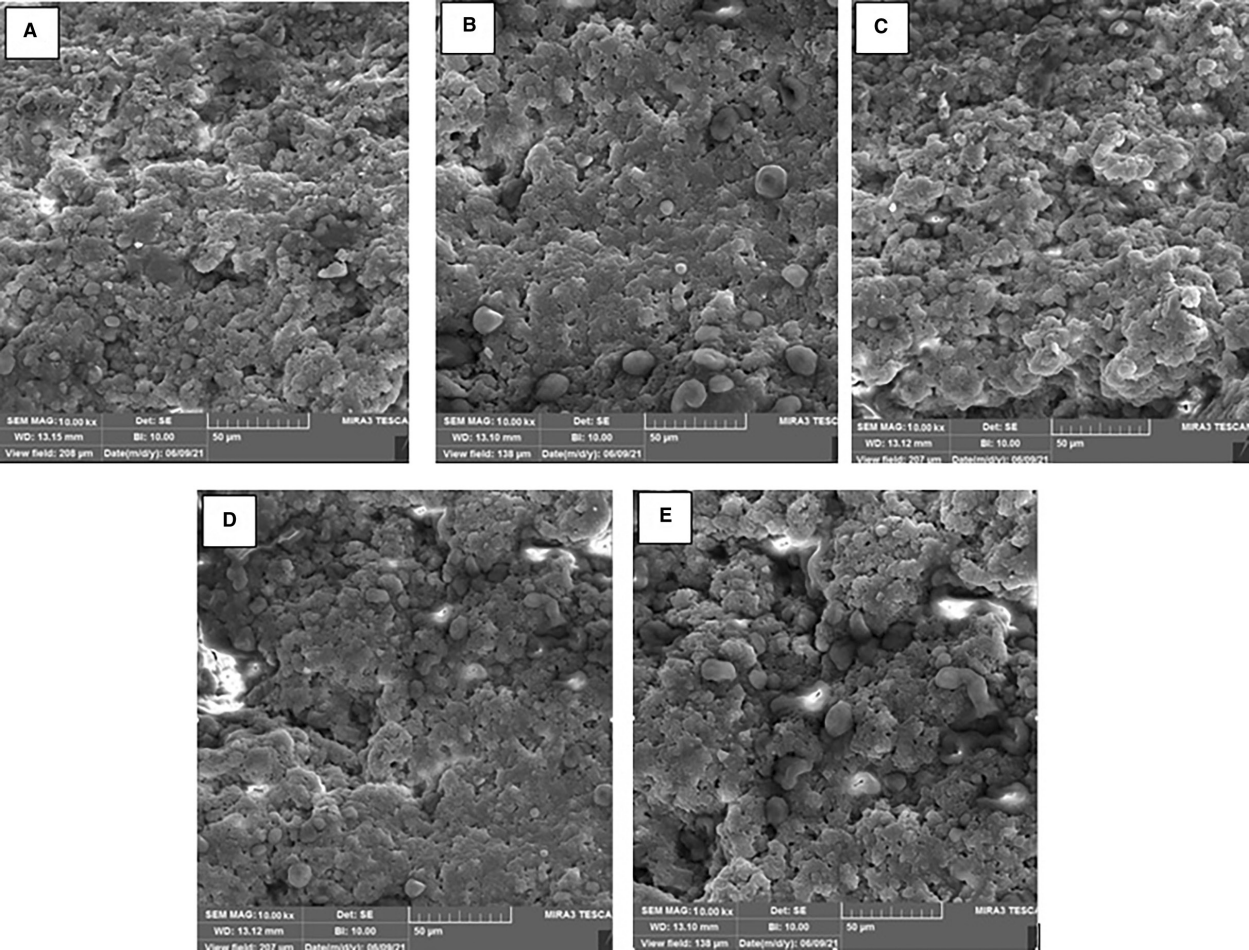
SEM micrographs of the UF‐white cheeses produced using different ratios of mesophilic starter culture and *L. helveticus* after 90 days of ripening (Cheeses A: 100% mesophilic starter culture; (B) 75% mesophilic starter culture + 25% *L. helveticus*; (C) 50% mesophilic starter culture + 50% *L. helveticus*; (D) 25% mesophilic starter culture + 75% *L. helveticus*; E: 100% *L. helveticus*).

The SEM images of the cheeses after 90 days of ripening are shown in Figure 3. According to the results, the type and ratios of starter cultures had an important effect on the porous structure of the casein within the matrix of the cheeses. As the *L. helveticus* ratio increased, the larger pores were visible in the UF‐white cheese microstructure. In this regard, the cheeses D and E that produced using higher ratios of *L. helveticus* had relatively larger pores and more evident spaces than the cheeses produced with higher ratios of mesophilic culture (cheeses A and B). Probably, higher proteolytic activity of *L. helveticus* compared to mesophilic starter culture (Baptista & Gigante, 2022; Sıçramaz et al., 2022) may cause higher proteolysis rate and softer texture in the cheeses D and E. On the other hand, the SEM images showed excellent accordance with the results of the rheology analysis. The cheeses A and B had a more compact casein network (Figure 2) and solid‐like viscoelastic structure (Figure 1) among the UF‐white cheeses, which might be attributed to less proteolytic activity of mesophilic starter culture compared with *L. helveticus* which resulted in less protein breakdown (Özer & Kesenkaş, 2019; Soltani et al., 2016).

### 3.6 Sensory evaluation

The results of the sensory evaluation of the UF‐white cheeses during ripening are shown in Table 4. Various combinations of two different cultures did not influence significantly the color and appearance of cheeses during ripening (*p* > .05). In terms of the structure and texture, using higher ratios of *L. helveticus* caused formation of a less compact protein network and a softer texture in UF‐white cheese (*p* < .05). In this context, Kumar et al. (2015) reported that hydrolysis of α_s1_‐CN reduced the hardness of Feta‐type cheese which is in accordance with the fewer points received by the cheeses produced using higher ratios of *L. helveticus* (cheeses D and E) in terms of the structure and texture.

**TABLE 4:** Sensory characteristics of UF‐white cheeses produced using different ratios of mesophilic starter culture and *L. helveticus* during ripening (*n* = 3).

	A*	B	C	D	E
Appearance and color					
1	5.00 ± 0.00^aA^	5.00 ± 0.00^aA^	4.88 ± 0.00^aA^	4.88 ± 0.00^aA^	4.88 ± 0.00^aA^
30	4.94 ± 0.06^aA^	4.94 ± 0.6^aA^	4.82 ± 0.05^aA^	4.77 ± 0.00^aA^	4.71 ± 0.05^aA^
60	4.94 ± 0.06^aA^	4.88 ± 0.11^aA^	4.77 ± 0.11^aA^	4.71 ± 0.05^aA^	4.71 ± 0.05^aA^
90	4.82 ± 0.05^aA^	4.82 ± 0.05^aA^	4.71 ± 0.05^aA^	4.66 ± 0.11^aA^	4.66 ± 0.11^aA^
Body and texture					
1	4.88 ± 0.00^aA^	4.82 ± 0.05^aA^	4.55 ± 0.11^bA^	4.49 ± 0.05^bcA^	4.27 ± 0.05^cB^
30	4.94 ± 0.06^aA^	4.94 ± 0.06^aA^	4.60 ± 0.16^bA^	4.55 ± 0.11^bA^	4.44 ± 0.11^cAB^
60	5.00 ± 0.00^aA^	4.94 ± 0.06^abA^	4.71 ± 0.05^bcA^	4.66 ± 0.11^cA^	4.60 ± 0.05^cA^
90	4.82 ± 0.05^aA^	4.82 ± 0.05^aA^	4.49 ± 0.05^bA^	4.44 ± 0.11^bA^	4.16 ± 0.05^cB^
Odor and flavor					
1	8.99 ± 0.11^cA^	9.05 ± 0.05^bcA^	9.11 ± 0.11^bA^	9.66 ± 0.11^aA^	9.71 ± 0.16^aA^
30	8.77 ± 0.11^cB^	8.94 ± 0.06^bAB^	8.94 ± 0.06^bB^	9.39 ± 0.11^aB^	9.44 ± 0.11^aB^
60	8.38 ± 0.5^dC^	8.60 ± 0.16^cBC^	8.77 ± 0.11^bC^	8.94 ± 0.06^aC^	9.00 ± 0.00^aC^
90	8.11 ± 0.11^dD^	8.38 ± 0.05^cC^	8.60 ± 0.05^bD^	8.76 ± 0.00^aD^	8.84 ± 0.11^aC^
Overall acceptance					
1	18.87 ± 0.16^aA^	18.87 ± 0.06^aA^	18.54 ± 0.00^bA^	19.03 ± 0.11^aA^	18.86 ± 0.06^aA^
30	18.65 ± 0.11^bcAB^	18.82 ± 0.20^aAB^	18.37 ± 0.17^dA^	18.71 ± 0.17^bA^	18.59 ± 0.27^cB^
60	18.32 ± 0.06^bBC^	18.42 ± 0.15^aB^	18.25 ± 0.05^cAB^	18.31 ± 0.11^bB^	18.31 ± 0.05^bC^
90	17.75 ± 0.22^cC^	18.02 ± 0.05^aB^	17.81 ± 0.16^bB^	17.86 ± 0.27^bB^	17.66 ± 0.27^dD^

*Note*: Means with different superscripts within each UF‐white cheese (upper case) and within each ripening time (lower case) for each of the parameters measured, are different (*p* < .05).

* Cheeses A: 100% mesophilic starter culture; B: 75% mesophilic starter culture + 25% *L. helveticus*; C: 50% mesophilic starter culture + 50% *L. helveticus*; D: 25% mesophilic starter culture + 75% *L. helveticus*; E: 100% *L. helveticus*.

Significant differences were observed in the odor and flavor of UF‐white cheeses during ripening. Utilization of higher ratios of *L. helveticus* for cheese production led to receiving significant higher odor and flavor scores in comparison to the cheeses made using higher ratios of mesophilic starter culture. In this context, it has been reported that more hydrolysis of caseins and production of flavor enhancement peptides led to flavor development in Cheddar, Swiss‐Type and Pasta Filata cheeses manufactured using *L. helveticus* as starter culture (Hannon et al., 2007; Sadat‐Mekmene et al., 2013; Sıçramaz et al., 2022). Finally, the score of overall acceptance in the cheeses indicated that the UF‐white cheeses produced using mesophilic starter culture and *L. helveticus* at the ratios of 75:25 and 25:75 had acceptance scores close and higher than the control cheese (cheese A) and can be suggested for production on industrial scale.

**FIGURE 4:**
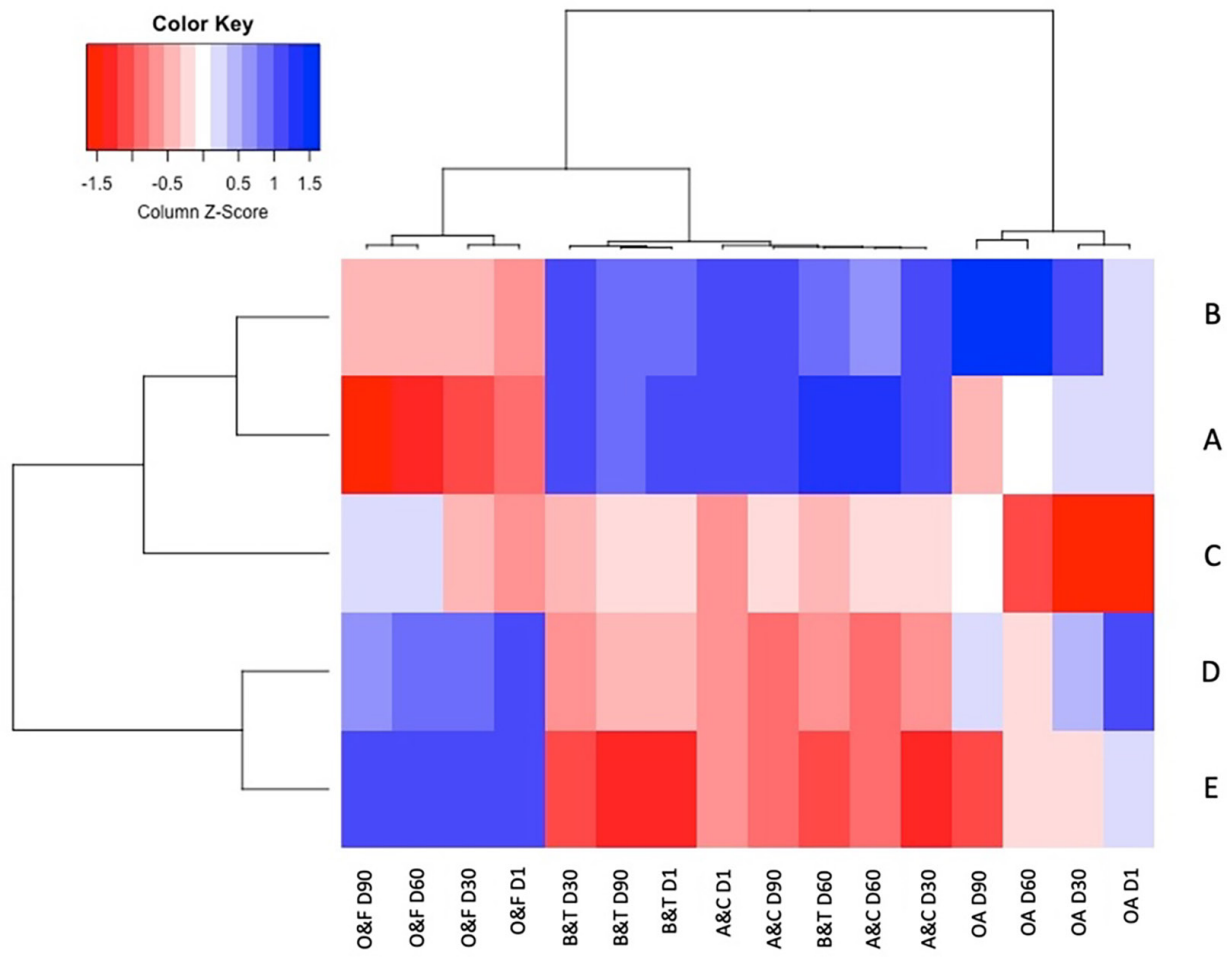
Heat map of sensory properties of UF‐white cheeses during ripening (Cheeses A: 100% mesophilic starter culture; B: 75% mesophilic starter culture + 25% *L. helveticus*; C: 50% mesophilic starter culture + 50% *L. helveticus*; D: 25% mesophilic starter culture + 75% *L. helveticus*; E: 100% *L. helveticus*). (A and C: appearance and color; B and T: body and texture; O and F: odor and flavor; OA: overall acceptance).

The sensory properties of UF‐white cheeses were also presented in a heat map (Figure 4). As seen, the cheeses have been clustered based on differences in the ratio of two types of starter culture used for their production. The first cluster represented the cheeses produced with higher concentrations of mesophilic starter culture (cheeses A and B), while the second cluster consisted of the cheeses made with higher ratios of *L. helveticus* (cheeses D and E). In this context and according to heat map, while higher scores of the structure and texture were assigned to the cheeses produced with higher ratios of mesophilic starter culture, the cheeses produced using higher levels of *L. helveticus* had higher scores of odor and flavor.

## 4 CONCLUSION

The different ratios of starter cultures used in this study had a considerable effect on the chemical composition, proteolysis, rheology, microstructure, and sensory characteristics of UF‐white cheese. Because of the more proteolytic activity of *L. helveticus* compared to mesophilic starter culture, the cheeses produced using higher ratios of *L. helveticus* had lower pH and protein contents and higher proteolysis and lipolysis values than the other cheeses. However, using higher ratios of *L. helveticus* caused to decrease in elastic behavior and formation of a more open structure in the final product. Based on sensory evaluation, texture of the cheeses produced using higher amount of *L. helveticus* had lower scores, although these cheeses received higher odor and flavor scores. The findings of the present study can help to a better understanding of the relationship between starter cultures used for UF‐white cheese production and the quality characteristics of the final product. As well, the outcomes of this study declare the result of acceleration of proteolysis in UF‐white cheese and producing a food product with developed texture and flavor.

## AUTHOR CONTRIBUTIONS

**Arash Tondhoush:** Formal analysis (equal); investigation (equal); writing – original draft (equal). **Mostafa Soltani:** Conceptualization (equal); methodology (equal); supervision (equal); writing – review and editing (equal). **Fatemeh Azarikia:** Data curation (equal); formal analysis (equal); methodology (equal); software (equal); writing – review and editing (equal). **Aziz Homayouni‐Rad:** Data curation (equal); visualization (equal); writing – review and editing (equal). **Mostafa Karami:** Data curation (equal); methodology (equal); visualization (equal).

## CONFLICT OF INTEREST STATEMENT

The authors declare that they do not have any conflict of interest.

## ETHICS STATEMENT

This study does not involve any human or animal testing.

## INFORMED CONSENT

Written informed consent was obtained from all study participants.

## Data Availability

The data that support the findings of this study are available from the corresponding author upon reasonable request.
